# Modification and Assembly of a Versatile Lactonase for Bacterial Quorum Quenching

**DOI:** 10.3390/molecules23020341

**Published:** 2018-02-06

**Authors:** Melissa K. Rhoads, Pricila Hauk, Valerie Gupta, Michelle L. Bookstaver, Kristina Stephens, Gregory F. Payne, William E. Bentley

**Affiliations:** 1 Institute for Bioscience and Biotechnology Research (IBBR), University of Maryland, College Park, MD 20742, USA; melissa.rhoads39@gmail.com (M.K.R.); pricila.hauk@gmail.com (P.H.); kstephe@terpmail.umd.edu (K.S.); gpayne@umd.edu (G.F.P.); 2 Fischell Department of Bioengineering, University of Maryland, College Park, MD 20742, USA; valerie12@gmail.com (V.G.); mlbooks@umd.edu (M.L.B.)

**Keywords:** quorum quenching, antibiotics, organophosphate, enzyme attachment, enzyme delivery

## Abstract

This work sets out to provide a self-assembled biopolymer capsule activated with a multi-functional enzyme for localized delivery. This enzyme, *Sso*Pox, which is a lactonase and phosphotriesterase, provides a means of interrupting bacterial communication pathways that have been shown to mediate pathogenicity. Here we demonstrate the capability to express, purify and attach *Sso*Pox to the natural biopolymer chitosan, preserving its activity to “neutralize” long-chain autoinducer-1 (AI-1) communication molecules. Attachment is shown via non-specific binding and by engineering tyrosine and glutamine affinity ‘tags’ at the C-terminus for covalent linkage. Subsequent degradation of AI-1, in this case *N*-(3-oxododecanoyl)-l-homoserine lactone (OdDHL), serves to “quench” bacterial quorum sensing (QS), silencing intraspecies communication. By attaching enzymes to pH-responsive chitosan that, in turn, can be assembled into various forms, we demonstrate device-based flexibility for enzyme delivery. Specifically, we have assembled quorum-quenching capsules consisting of an alginate inner core and an enzyme “decorated” chitosan shell that are shown to preclude bacterial QS crosstalk, minimizing QS mediated behaviors.

## 1. Introduction

The World Health Organization (WHO) has identified antibiotic resistance as “… one of the biggest threats to global health today. It can affect anyone, of any age, in any country” [1]. Despite this widespread health concern, few new antibiotics are being developed, and current antibiotics are losing effectiveness. As a result, new methods of combating bacteria are being explored, including interrupting or “quenching” bacterial communication pathways [2,3,4]—particularly those that lead to biofilms [5,6,7] or other more harmful behaviors. There are several classes of bacterial communication molecules that can be targeted, including homoserine lactones, *N*-Acyl homoserine lactones (AHLs), or autoinducers AI-1, which are used by Gram-negative bacteria such as *Pseudomonas aeruginosa* to control population based behavior. Enzymes that hydrolyze the ester bond of lactones, lactonases, have been shown to render the communication molecule unrecognizable to cells, disrupting the communication pathways [8,9,10,11]. Specifically, population-scale phenotypes can be modified by interfering with bacterial communication, providing a non-bactericidal means of altering bacterial behavior [12]. However, to quench AHL communication, many different molecules which are contained within the AHL family of autoinducers used by different bacteria, must be addressed. As a result, a lactonase that is capable of hydrolyzing several different AHLs is desirable and was a major contributing factor in choosing the enzyme used in this study.

The WHO has also identified chemicals of significant public health concern, including organophosphates (OP) that are active ingredients in pesticides [13]. OPs inhibit acetyl cholinesterase activity, which leads to decreased nerve function [14] and long-term developmental and behavioral dysfunction [15,16]. OPs have also been shown to affect liver, respiratory and cardiac function [16]. As a result, phosphotriesterases (PTEs), which neutralize organophosphates by hydrolysis of organophosphate esters [17], are of interest for treatment both prophylactically and after exposure [18,19].

**Figure 1:**
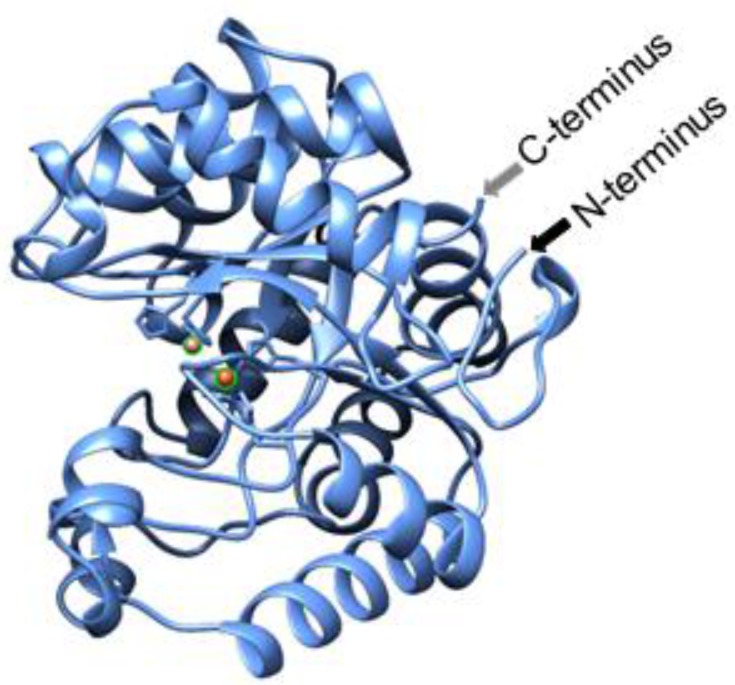
Cartoon of *Sso*Pox. This cartoon, generated using the Expasy Swiss-model (www.expasy.org) and then visualized using UCSF Chimera (www.cgl.ucsf.edu) with the same sequence as 2VC5, illustrates the orientation of the N- and C-termini as well as highlights the cobalt ions shown as orange balls at the location of the active site.

Because hydrolysis is required for neutralizing both classes of molecules (OP and AHL), an increasing number of studies have been conducted on enzymes which have both lactonase and PTE activity [18,20]. *Sso*Pox, named based on the organism of its origin, the hyperthermophilic *Sulfolobus solfactircus*, *Sso* [21], has such activity and was originally identified as a paraoxonase (Pox) [22,23]. It was chosen for this work as it exhibits promiscuous lactonase [24] activity with a preference toward AHLs with 8–10 carbon aliphatic chains and oxo-lactones with shorter chains [24] demonstrating non-specific hydrolysis [25]. The enzyme can be produced in *E. coli*, has high thermostability [21] and has a structure wherein the active site is opposite the N- and C-termini, as seen in Figure 1. These structural characteristics led to our hypothesis that *Sso*Pox activity could be maintained when the protein is attached to a surface via its termini. Thus, we modified *Sso*Pox for assembly onto the biopolymer, chitosan, in order to enable its localization for various applications, using AHL hydrolysis as the exemplar in this study.

In this work, three tags have been added to modify *Sso*Pox: a hexa-histidine tag has been added to the N-terminus and two different tags, respectively, have been added to the C-terminus, either a penta-tyrosine tag or quaternary glutamine tag. The hexa-histidine tag provides for facile purification using a charged immobilized metal ion affinity chromatography (IMAC) column-previously, many steps including heating, H/F-PLC, NaCl gradients, and dialysis were used in *Sso*Pox purification [22,26]. The tyrosine and glutamine tags have been added to the C-terminus to facilitate covalent binding to the biopolymer chitosan [27,28]. These were specifically chosen based on success in maintaining activity of other proteins after attachment to chitosan [27,28,29,30] and more widespread use of free-amine binding for functionalizing surfaces [31].

## 2. Results

By attaching *Sso*Pox to the biopolymer chitosan, which, in turn, can be fabricated into several forms [27,32] and is used in drug delivery and wound healing applications [33], we may be able to provide new and innovative ways to deliver its lactonase and potentially, organophosphate hydrolase activity to various sites, including in humans. Here, lactonase activity is verified for the AI-1, *N*-(3-oxododecanoyl)-l-homoserine lactone (OdDHL), which stimulates inflammation in mammalian cells and promotes production of the toxin pyocyanin of *P. aeruginosa* [34].

### 2.1. Purification of Modified SsoPox

**Figure 2:**
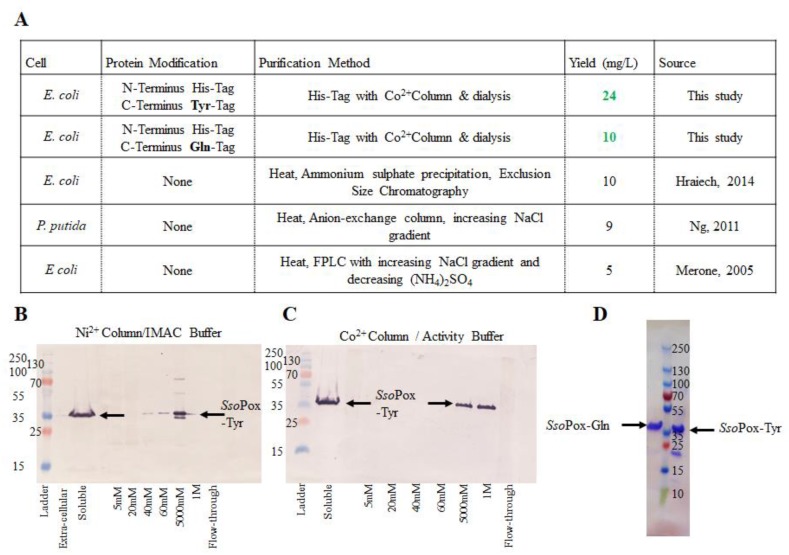
Purified *Sso*Pox-Tyr/Gln. A summary of the construction, purification and yield of the enzymes developed in this work are compared to previous work in (**A**). (**B**,**C**) depict representative Western blots comparing purification profiles of Ni^2+^ (**B**) vs. Co^2+^ (**C**) IMAC columns. In (**D**) the purified samples of each *Sso*Pox-Gln (left of ladder) and *Sso*Pox-Tyr (right of ladder) on an SDS-PAGE gel can be found.

*Sso*Pox was first modified with a hexa-histidine tag, enabling simple and rapid purification via IMAC. Successful results depicted in Figure 2A also include purification and yields from previous studies. Our tyrosine-modified *Sso*Pox, *Sso*Pox-Tyr, was initially purified using a Ni^2+^-loaded IMAC column. While the IMAC elution yielded enzyme as shown in the Western blot in Figure 2B, the activity buffer (AB) did not, likely due to ionic competition between the Ni^2+^ column and the Co^2+^ in the buffer (not shown). To avoid divalent cation interference and recognizing that *Sso*Pox is a metalloenzyme with cobalt as the stabilizing metal ion, a Co^2+^ IMAC column was used in further work. This yielded a “clean” eluate; a Western blot is shown in Figure 2C. Correspondingly, *Sso*Pox-Tyr was sequentially rinsed with 20 mM and 60 mM imidazole in AB before final elution with 1 M imidazole. Similar experiments with a Co^2+^ column and increasing concentrations of imidazole in AB were conducted for *Sso*Po*x*-Gln (not shown). *Sso*Pox-Gln purification was successful as rinsed with 40 mM and 100 mM imidazole in AB before elution with 300 mM imidazole. A representative SDS-PAGE gel depicting the elution of each *Sso*Pox variant is shown in Figure 2D.

### 2.2. AI-1 Reporters

Three *E. coli* reporters (see Methods) were used to indicate AI-1 levels in solutions. These were based on LasR-mediated gene expression [35,36,37]. A linear relationship between AI-1 concentration and luminescence is shown in Figure S1. Analogously, the AI-1 response of two fluorescent reporters measured using flow cytometry is shown in Figure S2 where the linear ranges of these reporters are provided.

### 2.3. SsoPox Quorum Quenching

**Figure 3:**
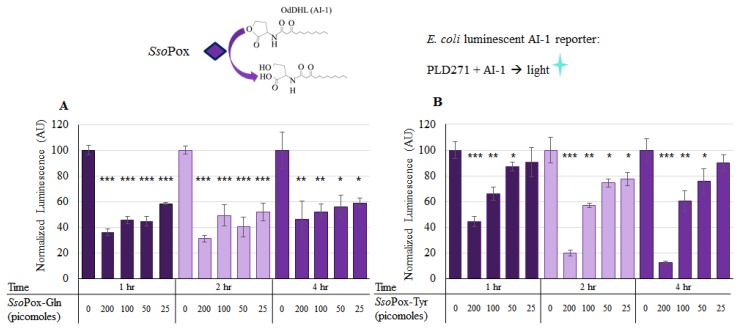
AI-1 quenching with lactonase in solution. In (**A**), known quantities of *Sso*Pox-Gln were incubated in solution with 225 µM AI-1 (OdDHL) and aliquots of each of three samples were taken at 1, 2 and 4 h. Similarly, in (**B**), known quantities of *Sso*Pox-Tyr were incubated with 225 µM AI-1 and aliquots of each of three samples were taken at 1, 2 and 4 h. These samples were then diluted 1000× and measured for relative OdDHL concentration using the JLD271 cell lines; with higher concentrations of OdDHL producing more light/luminescence (See Figure S2). The results are the average of triplicate samples with standard deviations, *p*-values are calculated using a Student’s *t*-Test, two-tailed, type 2; * *p* < 0.05, ** *p* < 0.008, *** *p* < 0.0008.

Using the luminescent reporter (Lindsay and Ahmer [38]), the activity of the two modified forms of *Sso*Pox were verified in solution. Both *Sso*Pox-Gln and *Sso*Pox-Tyr were tested for activity in a final solution of 100 µL with 225 µM OdDHL (AI-1) incubated at 37 °C with shaking for up to 4 h. Varying levels of *Sso*Pox, from 0 to 200 pmol, were added as indicated in Figure 3. In Figure 3a, the first five columns/bars show the AI-1 activity after the samples were incubated for 1 h with *Sso*Pox-Gln at various levels. Here, it can be seen that with 25, 50, 100, and 200 pmol, there was a significant decrease in AI-1 activity (Student’s *t*-test *p*-value < 0.0008). The same was true for the 2 h sample, with all experimental samples exhibiting statistically significant decreases in AI-1 activity. At 4 h, statistical significance was similarly maintained. Interestingly, in Figure 3b, *Sso*Pox-Tyr demonstrated a significant decrease after just 1 h for the 100 and 200 pmol samples, but little decrease when 25 pmol were present, even after 4 h.

The difference in activity of the two forms of *Sso*Pox could be the result of many factors and additional research is needed to attribute altered activity to the presence or absence of the Tyr or Gln tags. Nonetheless, these data indicate that both variants of *Sso*Pox exhibited activity; the next step was to verify the ability to couple the two variants to chitosan.

**Figure 4:**
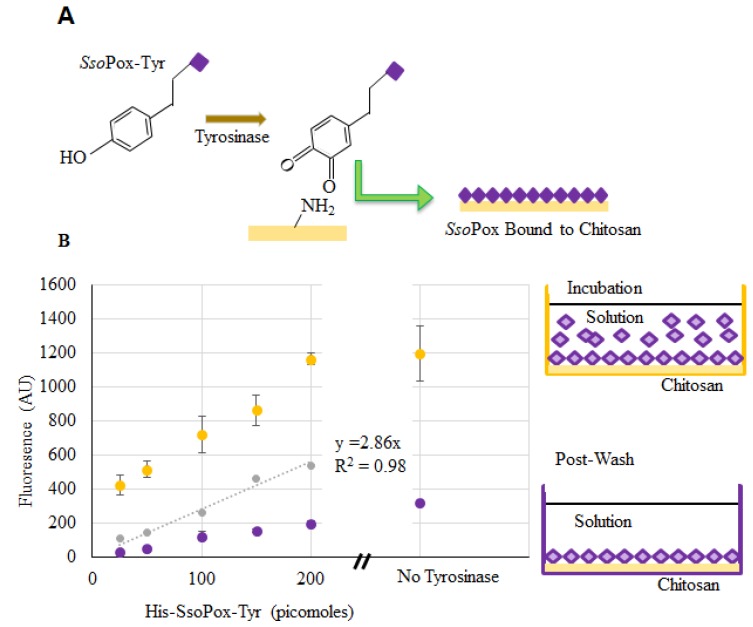
*Sso*Pox-Tyr binding to chitosan. In (**A**), the binding of *Sso*Pox-Tyr is illustrated using tyrosinase to form an *o*-quinone on the tyrosine residue which covalently links the amine groups on chitosan; (**B**) Fluorescent measurements taken at the completion of incubation (yellow) and again after washes (purple). Points indicated are the average of 3 samples, error bars are the standard deviation. The gray dataset represents a standard curve for the quantity of bound labeled enzyme.

Verification of *Sso*Pox binding to chitosan films and capsules was completed using enzyme labeled with DyLight^TM^ Sulfhydryl-Reactive Dye (ThermoFisher, Waltham, MA, USA), which reacts primarily with free –SH groups, such as those found on cysteine. Chitosan films in the bottom of a 96-well plate were used to verify binding and estimate binding density. Labeled *Sso*Pox-Tyr was incubated with the dried chitosan and tyrosinase in solution. The tyrosinase modifies the tyrosyl residues of *Sso*Pox-Tyr to *o*-quinones which subsequently bind to the primary amines of chitosan [27,29,30] as illustrated in Figure 4a. Fluorescence measurements were taken from wells incubated with various quantities of labeled *Sso*Pox-Tyr. After incubation, the wells were washed and additional readings were taken (represented by the “Post-Wash” illustration). At the same time, fluorescence of samples with known quantities of labeled but unbound *Sso*Pox-Tyr were taken to create a calibration model and to estimate the quantity of bound *Sso*Pox-Tyr when incubated with ~150–200 pmol [39]. We subsequently estimated that the quantity of *Sso*Pox-Tyr bound to chitosan when incubated with 200 pmol was ~60 pmol. This quantity is far greater than that estimated assuming a uniformly packed monolayer of protein assembled onto the bottom of a flat well (~2 pmol [39]).

In addition to the samples with tyrosinase, there were three samples incubated without tyrosinase (200 pmol *Sso*Pox-Tyr). These were washed in the same manner and fluorescence was recorded. Interestingly, our data (Figure 4b) suggest that more enzyme (~110 pmol) was bound non-covalently/nonspecifically than when coupled via tyrosinase-conjugation chemistry. We have recently reported that the tyrosinase itself binds non-covalently to the chitosan, thereby “competing” for binding sites in experiments with *Sso*Pox when tyrosinase is present [28]. Also, there exists the possibility that tyrosinase facilitates some enzyme oligomerization effecting the quantity bound (or measured as bound) [40,41,42]. Perhaps more importantly, *Sso*Pox remained bound even after washing. Strong nonspecific binding via charge interactions have been noted before [26].

**Figure 5:**
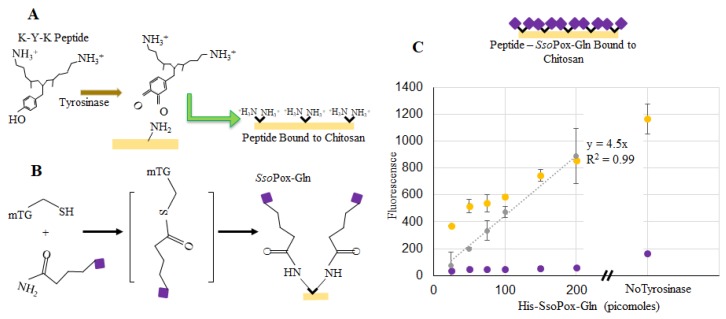
*Sso*Pox-Gln binding to chitosan. In (**A**), the binding of the KYK peptide is illustrated using tyrosinase to form an *o*-quinone on the tyrosine residue which binds to the primary amine groups on chitosan; (**B**) Shows the multi-step process whereby microbial transglutaminase (mTG) aids in binding the *Sso*Pox-Gln to the lysine groups of the peptide (**C**) indicates fluorescence readings at the completion of incubation (yellow) and again after washes (purple). Points represent the average of 3 points; error bars are the standard deviation. The gray line is a linear best-fit for the calculated bound enzyme.

Similarly, binding of *Sso*Pox-Gln was tested as fluorescently labeled. Figure 5a,b illustrates a two-step binding process using tyrosinase first and then transglutaminase [40]. Specifically, this approach uses tyrosinase to covalently couple lysine-tyrosine-lysine (KYK) peptides to the primary amines of chitosan. In this way, the chitosan is “prepared” for coupling to the *Sso*Pox through a glutamine tag. We use a microbial transglutaminase to link the glutamines attached to the protein terminus to the peptide lysine amines coupled to the chitosan. We found this method to be quicker and with less nonspecific binding than the one-step tyrosine approach [40]. Figure 5c depicts the average fluorescence of three samples with known quantities of labeled *Sso*Pox-Gln before and after washing each well three times, with error bars representing the standard deviations. A standard curve was again created using known concentrations of labeled *Sso*Pox-Gln and this curve was used to calculate the amount of bound *Sso*Pox-Gln in 200 pmol samples after washing (~12 pmol). In addition to verifying binding via the two-step process for the *Sso*Pox-Gln, 200 pmol samples were again incubated without transglutaminase and ~35 pmol were found to remain bound after washing.

As noted, we estimated that *Sso*Pox-Tyr (~60 pm w/tyrosinase, ~110 w/o tyrosinase) and *Sso*Pox-Gln (~12 pm w/transglutaminase, ~35 pm w/o transglutaminase) were assembled onto the chitosan, after washing, at the bottom of 96-well plates when incubated with 200 pmol in solution. Conversely, we estimated that a *single layer* of either *Sso*Pox-Tyr or *Sso*Pox-Gln would comprise ~7.6 pmol/cm^2^, or 1.9 pmol total. To make this calculation we made projected area-based estimates of a monolayer of protein (sphere) on a flat surface (chitosan) [39]. This took into consideration closest circle packing densities for a coverage of 78%, and did not consider repulsion of the similarly charged molecules. Because the enzymes can aggregate and the chitosan surface is not flat, these estimates effectively represent the minima if the entire surface is covered. Our experimental results indeed demonstrate that more enzyme was bound than predicted. That is, it is likely that labeled SsoPox was bound within the matrix of chitosan, rather than just on top of a flat surface. While the levels of assembled protein were ~6 to 50-fold more than what would exist as a single layer, the thickness of such protein layers assuming that the protein binds within the chitosan matrix would comprise only 0.1–0.3% the available chitosan- a value significantly thinner than the chitosan films.

**Figure 6:**
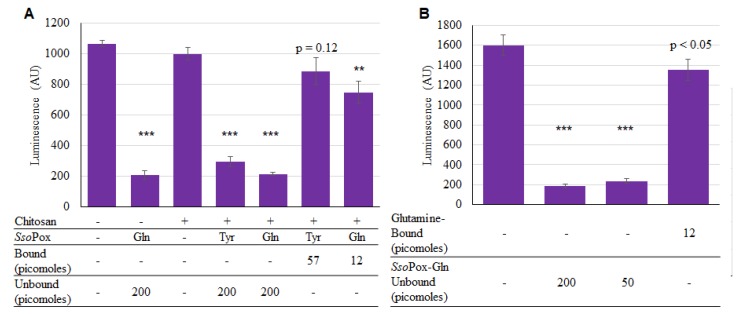
Activity of bound *Sso*Pox-Tyr/Gln. In (**A**), 200 pmol of unbound *Sso*Pox, and 57 and 12 pmol of *Sso*Pox-Tyr and *Sso*Pox-Gln, respectively were bound; samples were incubated for 2 h at 37 °C with 225 µM AI-1 solution; In (**B**), only *Sso*Pox-Gln was tested and samples were incubated 1 h, again at 37 °C with 20 µM AI-1 solution. In both charts the bars are the average of three samples with standard deviations provided. *p*-values are calculated by Student’s *t*-Test, two tails, type 2; ** *p* < 0.008, *** *p* < 0.0008.

Thus, both *Sso*Pox-Tyr and *Sso*Pox-Gln were successfully bound onto the chitosan surfaces. While *Sso*Pox-Gln’s enhanced activity in solution may have suggested proceeding without *Sso*Pox-Tyr, we still wanted to test activity of both versions while bound. As described, chitosan films in 96-well plates were incubated for 2 h with enzymes and unbound *Sso*Pox-Gln was included as a positive control. As seen in Figure 6a, both unbound enzymes were active, demonstrating statistically significant decreases in AI-1 activity. However, for the bound samples, the bound *Sso*Pox-Gln provided more of a decrease in AI-1 concentration than *Sso*Pox-Tyr. In Figure 6b it was evident that there was a small difference in activity between the two amounts of unbound *Sso*Pox-Gln (200 vs. 50 pmol), as expected given previous results. In summary, it was apparent that *Sso*Pox-Gln was superior as a quorum quencher under these conditions; it was subsequently used for assembly onto capsules.

### 2.4. SsoPox-Gln Capsules for Quorum Quenching

**Figure 7:**
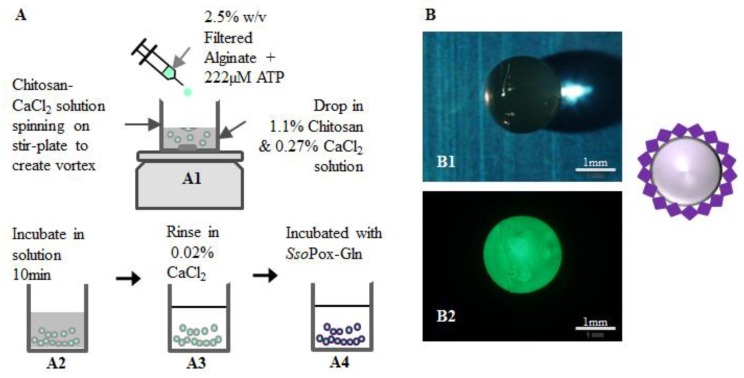
Capsule construction for AI-1 lactonase quenching. As illustrated in (**A**), capsules were constructed by dropping a mixture of 2.5% *w*/*v* filtered alginate and 222 µM ATP via 27 G needle into a stirred chitosan-CaCl_2_ solution to form capsules (**A1**) which were then incubated 10 min (**A2**). The capsules were removed from the incubation media and rinsed in 0.02% *w*/*v* CaCl_2_ (**A3**) before binding with *Sso*Pox (**A4**) and subsequent QS quenching. Images of the capsules as taken by a stereo-microscope are shown (**B1**,**B2**). Here green fluorescently-labeled *Sso*Pox-Gln is bound to a capsule.

Using the identical components (e.g., enzymes and chitosan), we constructed capsules that could be more easily deployed into solutions of varied origin. For this, we used an additional natural polysaccharide, alginate. The assembled capsules ultimately consist of an alginate inner core and a chitosan outer shell to which the enzymes are bound. Construction of the functionalized capsules is based on the polyelectrolyte character of chitosan and alginate bilayers enabling their self-assembly [43]. While layer-by-layer systems have been constructed, we used a one-step procedure [39]. ATP (10 mM) was mixed with alginate (2.5%) for final concentration of 222 µM ATP, vortexed, and added drop-wise by syringe needle (27 G) to a magnetically-stirred solution of 1.1% (*w*/*v*) chitosan and 0.27% (*w*/*v*) CaCl_2_. The alginate-chitosan capsules were left in solution for 10 min before removal and rinsing in 0.02% (*w*/*v*) CaCl_2_. These capsules (Figure 7) were then transferred to flasks for enzyme attachment. The outer diameter (1.375 mm) and shell thickness of capsules (0.2 mm) were measured immediately after rinsing using an MVX10 MacroView (Olympus, Center Valley, PA, USA) fluorescence stereomicroscope. Figure 7 illustrates the construction procedure and shows stereomicroscope images of green-labeled *Sso*Pox-Gln bound to the outside of the capsule. Note here that *Sso*Pox-Gln was non-specifically/non-covalently bound so as to provide enhanced quantities over the transglutaminase method. To bind the *Sso*Pox-Gln non-specifically, similar steps were taken as for covalent binding: capsules were incubated with the enzyme for 1 h at 37 °C with shaking, then the capsules were rinsed three times with HEPES pH 7.0. Fluorescence measurements revealed that capsule fluorescence was not statistically different than the earlier multi-well experiments, as seen in Figure S3. This was anticipated, as significant effort was expended to employ similar conditions between well and capsule experiments (e.g., quantities of protein, chitosan, fluid levels, etc.). As a result, slightly higher quantities of *Sso*Pox-Gln were bound per surface area of the capsule (146 pmol/cm^2^) than the wells (109 pmol/cm^2^). Even with this slightly higher degree of binding per surface area, an estimated enzyme layer (4.5 × 10^-7^ cm) was still less than 0.1% of the chitosan layer of the capsule (0.2 mm). We indicate this to suggest that mass transfer limitations of substrate and product species should be limited.

**Figure 8:**
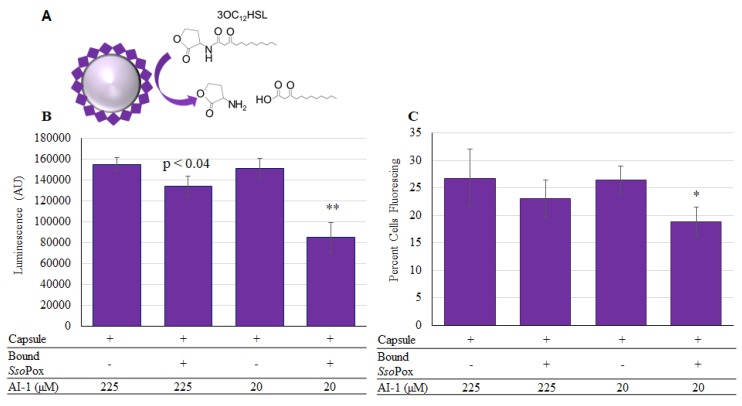
*Sso*Pox-Gln capsules assembled for AI-1 quenching and activity. In (**A**) the reaction in which *Sso*Pox-Gln hydrolyzes OdDHL is illustrated. In (**B**) the luminescent reporter is used to measure remaining AI-1 activity after samples were incubated for 2 h at 37 °C. In (**C**), the same samples were measured with the red-green fluorescent reporter and the chart reflects the percent of cells fluorescing as counted from a total of 50,000 using flow-cytometry. In both charts the bars are the average of three samples with standard deviations provided. *p*-values are calculated by Student’s *t*-Test, two tails, type 2; * *p* < 0.03, ** *p* < 0.008.

Owing to the increased level of attached enzyme, we used this non-specific binding technique in neutral pH, where the negatively charged *Sso*Pox-Gln (pI 6.28), is bound to the outside layer of chitosan [44], to create functional capsules. These were tested for their ability to quench the AI-1 mediated communication. As shown in Figure 8, capsules were incubated at two different concentrations of AI-1 and aliquots of the solution were taken after 2-h incubation at 37 °C. These same aliquots were measured using both the luminescent (Figure 8B) and the Red-Green fluorescent (Figure 8C) reporters. In the case of Red-Green reporters, the relative level of QS activity is indicated by the fraction of the indicator cells that express green fluorescent protein [45]. Importantly, *Sso*Pox-Gln was shown to reduce AI-1 activity in all samples when present. Interestingly, the biologically relevant concentrations for altering *P. aeruginosa* phenotype have been reported in the pico and nanomolar range [46,47,48,49], but reducing OdDHL (AI-1) concentrations to below 70 µM has been shown to enable stimulated immune function in infected mice [49]. That is, mice infected with *P. aeruginosa* that exhibit high levels of OdDHL are immune compromised and by reducing this level (indicating fewer *P. aeruginosa*) immune function is restored. Hence, the activities and concentrations used in these studies are biologically relevant. In addition, it is important to indicate that while we have only tested OdDHL in this study, SsoPox has been shown to be active for several different AHLs [24].

## 3. Discussion

All modified *Sso*Pox enzymes retained lactonase activity in solution. Importantly, *Sso*Pox-Gln demonstrated significant reduction of AI-1 activity and therefore lactonase activity, when bound to chitosan. The addition of histidine, tyrosine, and glutamine tags to the N- and C-termini of *Sso*Pox has provided new opportunities for purification and function/delivery. That is, the histidine tag enabled more simple purification and opportunities for yield improvement. The tyrosine tag enabled direct covalent attachment to the primary amines of chitosan via tyrosinase. The glutamine tag enabled covalent attachment to chitosan that had been pretreated with tyrosine/lysine peptides. Specifically, a microbial transglutaminase links the glutamine of *Sso*Pox to the amines of lysine. We found the glutamine-tagged *Sso*Pox when assembled onto chitosan in multiwell plates and on capsules was most effective in eliminating AI-1 activity in fluids. While not tested in the GI tract of humans or mice, the biofabrication methodology presented here for “device” assembly demonstrates that *Sso*Pox can be modified, assembled, and delivered for reducing autoinducer-mediated QS activity. That is, it is particularly noteworthy that the assembly of proteins onto Nature’s polysaccharides, chitosan and alginate, is made via simple non-specific binding or via natural enzymatic conjugation. All components are of biological origin and are assembled via biologically benign methods. Thus, the strategies developed here are likely to preserve activity during “device” assembly and preclude pleotropic effects in various applications, such as human health, due to materials associated with construction and/or delivery.

## 4. Materials and Methods

### 4.1. SsoPox Expression Plasmids

The *Sso*Pox genetic sequence was optimized for *E. coli* using the IDT codon optimization tool and the gBlock and primers for insertion into pET200 plasmid were ordered from IDT (Coralville, IA, USA). The primer sequences and the gBlock sequence are found in Figure S3.

To construct the tyrosine-tagged *Sso*Pox, the gBlock was amplified using F-*Sso*Pox and *Sso*PoxR-Tyr. The purified PCR product was digested with SacI and NheI for sticky-end ligation into the pET200 (Invitrogen, Waltham, MA, USA) backbone to create pH*Sso*PoxTyr. To add the glutamine tag the pH*Sso*PoxTyr plasmid was digested with NheI and SacI. The *Sso*Pox gene was amplified with Q5 (NEB, Ipswich, MA, USA) using the same forward primer as used previously and a new reverse primer, *Sso*PoxR-Gln was used to add the glutamine tag. This PCR product was digested and ligated into the previously digested backbone. Sequences of the resultant plasmids (pH*Sso*PoxT and pH*Sso*PoxG) were verified by Genewiz (Frederick, MD, USA). After transformation into *E. coli* BL21(DE3) pLysS cells, the enzymes *Sso*Pox-Tyr (MW: 39.694 kDa) and *Sso*Pox-Gln (MW: 38.719 kDa) were expressed and purified.

### 4.2. Overexpression and Purification of Modified SsoPox

Cells were inoculated from frozen stock and grown overnight in LB (Fisher, Pittsburgh, PA, USA) supplemented with 50 µM kanamycin at 37 °C, 250 rpm. These cells were re-inoculated to OD_600_ 0.05 in 200 mL ZYP-5052 media without metals mix (Teknova, FisherScientific, Waltham, MA, USA. Cells were initially grown at 37 °C with shaking (250 rpm) until the culture reached OD600 1.0 (3.5–4 h). The 200 mL of culture in an Erlenmeyer flask was then subjected to cold-shock–swirled in ice-water for approximately 5 min—during which 0.2 mM CoCl_2_ was added as previously reported [23]. Cell growth continued at room temperature (~24 °C) for 20 h with shaking (250 rpm). Cells were then pelleted (10,000 g, 4 °C, 10 min), and re-suspended in a lysis buffer similar to that used by Hiblot et al. (50 mM HEPES pH 8, 150 mM NaCl, 0.2 mM CoCl_2_, 0.1 mM PMSF and 20 mM MgSO_4_) [23] and frozen at −80 °C. A Fisher Scientific 550 Sonic Membrator (Pittsburgh, PA, USA) at power 3.5 for 10 min 0.5 on/0.5 off was used to disrupt the membrane and cell debris was removed by centrifugation (10,000× *g*, 4 °C, 10 min).

Protein purification was achieved using a GE Healthcare Life Sciences HiTrapTM Chelating HP Column (Pittsburgh, PA, USA), and the hexa-histidine-tag on the *Sso*Pox-Tyr/GLn. Two columns were used in this work: one loaded with Ni^2+^, and the other loaded with Co^2+^. First, IMAC Buffer (50 mM sodium phosphate, 1.25 M NaCl, pH 7.4) was used for both columns, then the Activity Buffer described by Hiblot et al. [23] was used, both with increasing concentrations of imidazole after protein loading to determine optimal purification conditions. The purest sample of *Sso*Pox-Tyr/Gln underwent dialysis in Activity Buffer with gentle stirring at 4 °C until final imidazole concentration was less than 100 nM. The dialyzed enzyme concentration was measured using a Nanodrop and appropriate MW/Extinction coefficients (ThermoFisher, Waltham, MA, USA) (*Sso*Pox-Tyr: 39.7 kDa/37.4k, *Sso*Pox-Gln: 38.7 kDa/30k). They were stored in 10% sterilized glycerol at −20 °C.

### 4.3. AI-1 Reporter Construction

Construction of an AI-1 reporter was completed using constructs from Lindsay et al. [38], ‘standard parts’, a constitutive promoter developed in-lab and the pET21a backbone. This reporter, using genes and proteins from *P. aeruginosa*, produces green fluorescent protein in the presence of the AI-1, OdDHL. All primers were obtained from IDT (Coralville, IA, USA). Plasmid maps of the reporters are found in Figure S4. 

The first plasmid constructed, pAHL-Reporter_Red-Green, constitutively expresses dsRedExpress2 and *P. aeruginosa* LasR. LasR binds to OdDHL to form the OdDHL-LasR complex, which binds to the *P. aeruginosa* DNA binding site also incorporated into this plasmid. This DNA binding site is a positive transcriptional regulator that is activated upon binding of the OdDHL-LasR complex to the DNA, which activates transcription of sfGFP to signal the presence of OdDHL. This plasmid was transformed into the *E. coli* strain W3110 LuxS^−^ which does not produce AI-2. Subsequently, modifications were made to the plasmid using the restriction enzyme, BstEII, to cut 200 bps from the center of dsRedExpress2 from pAHL-Reporter_Red-Green, rendering the quaternary protein inactive, but maintaining LasR production, and forming a new plasmid: pAHL-Reporter_Green. The new plasmid was transformed into Top10 cells. Both reporter cells were stored as frozen stock until the day before use.

### 4.4. Determining Lactonase Activity of Modified SsoPox

The modified *Sso*Pox enzyme activity was verified using two reporter cells indicating the presence of AHL *N*-dodecanoyl-l-homoserine lactone (OdDHL). This AHL, in turn, was obtained from Cayman Chemical (Ann Arbor, MI, USA). The first reporter cell, a luminescent reporter, developed and provided by the Ahmer Lab [38] provides a quantitative indication of OdDHL activity in the nanomolar range. The second, developed here, uses the same *P. aeruginosa* LasR and DNA binding site, but includes a GFP fluorescent marker.

Incubation of the enzyme with OdDHL was completed in multi-well plates that included all controls. As a result, all samples were incubated for identical times and temperatures. They were completed in triplicate. Sample aliquots were removed and diluted for both luminescence and fluorescence-based assays.

The luminescent reporter was inoculated and grown overnight in LB with 50 µM kanamycin and 5 µM tetracycline at 37 °C, 250 rpm. Cells from the overnight culture were diluted 1:2500 µL in LB with 50 µM kanamycin and 5 µM tetracycline and 90 µL of diluted cells were added to 10 µL of the diluted incubation sample in a 5 mL test tube or 90 µL of cells were added to 10 µL of diluted sample in a 96-well white plate. The sample was incubated at 30 °C, 250 rpm for several hours and luminescence was measured using a GloMax^®^-Multi Jr (Promega, Madison, WI, USA) or Synergy HT plate reader (Fitchburg, MA, USA).

The fluorescent reporter cells were also inoculated and grown overnight in LB (100 µM ampicillin/carbenicillin at 37 °C, 250 rpm). These cells were re-inoculated and grown to OD_600_ 0.4 and again 90 µL of cell culture was added to 10 µL of the diluted incubation sample in a 5 mL test tube. This culture was incubated at 37 °C, 250 rpm, for 3 h and the percent of fluorescing cells was counted by flow cytometry.

### 4.5. Binding SsoPox-Tyr to Chitosan

*Sso*Pox-Tyr was constructed with a penta-tyrosine tag on the C-terminus for binding the protein to a surface containing primary amines. Methods similar to Wu et al. [27] were used, where 1.5% chitosan (Sigma, St. Louis, MO, USA) was dried overnight by vacuum incubation at 30 °C in a 96-well plate, then neutralized with 1 M NaOH and rinsed with HEPES pH 7.0 (Sigma, St. Louis, MO, USA). *Sso*Pox-Tyr was added in varying concentrations to 350 U of tyrosinase (Sigma, St. Louis, MO, USA) and HEPES pH 7.0 added to a final volume of 100 µL per well. After allowing the tyrosinase and *Sso*Pox-Tyr to incubate for 1 h at 37 °C with shaking, each well was rinsed with HEPES pH 7.0 three times, as previously described. Binding to chitosan films and capsules was verified by labeling *Sso*Pox-Tyr with DyLightTM Sulfhydryl-Reactive Dye (ThermoFisher Scientific, Grand Island, NY, USA). Bound His-*Sso*Pox-Tyr activity was verified by binding the protein as described, then repeating the methods described previously for protein activity.

### 4.6. Binding SsoPox-Gln to Chitosan

*Sso*Pox-Gln was constructed with a quaternary-glutamine tag on the C-terminus for transglutaminase-mediated binding to a surface. Methods similar to Bhokisham et al. [28] were used. Here a 60 µL 1 mM KYK peptide (Sigma, St. Louis, MO, USA) in pH 7.0 HEPES was combined with 350 u of tyrosinase and HEPES pH 7.0 was added to a total final volume of 100 µL. After allowing the peptide and tyrosinase to incubate for 1 h at 37 °C with shaking, each well was rinsed with HEPES pH 7.0 three times, as was done in previously. In this way, the tyrosine residue is used to confer lysines onto the chitosan.

Next, the *Sso*Pox-Gln was bound to the peptide using microbial transglutaminase (MTG) (Sigma, St. Louis, MO, USA). Here, MTG was prepared by making a 5% *w*/*v* solution in 10 mL of pH 7.0 HEPES and filtering this solution with a 0.22 µm filter. Final concentration of the MTG solution was measured using a Nanodrop^TM^ (ThermoFisher, Waltham, MA, USA). 60 µM of MTG was used to bind varying concentrations of *Sso*Pox-Gln to the peptide with HEPES pH 7.0 supplementing the solution to 100 µL; again incubation of binding occurred for 1 h at 37 °C with shaking. Binding to chitosan films and capsules was again verified by labeling *Sso*Pox-Gln with DyLightTM Sulfhydryl-Reactive Dye (ThermoFisher, Waltham, MA, USA). Bound *Sso*Pox-Gln activity was verified by binding the protein as described, then repeating the methods described previously for protein activity.

Next, the *Sso*Pox-Gln was bound to the peptide using microbial transglutaminase (MTG) (Sigma, St. Louis, MO, USA). Here, MTG was prepared by making a 5% *w*/*v* solution in 10 mL of pH 7.0 HEPES and filtering this solution with a 0.22 µm filter. Final concentration of the MTG solution was measured using a Nanodrop^TM^ (ThermoFisher, Waltham, MA, USA). 60 µM of MTG was used to bind varying concentrations of *Sso*Pox-Gln to the peptide with HEPES pH 7.0 supplementing the solution to 100 µL; again incubation of binding occurred for 1 h at 37 °C with shaking. Binding to chitosan films and capsules was again verified by labeling *Sso*Pox-Gln with DyLightTM Sulfhydryl-Reactive Dye (ThermoFisher, Waltham, MA, USA). Bound *Sso*Pox-Gln activity was verified by binding the protein as described, then repeating the methods described previously for protein activity.

### 4.7. Capsule Construction Materials

Alginate solution 2.5% (*w*/*v*) was made by dissolving medium viscosity alginate from brown algae (Sigma, St. Louis, MO, USA) in deionized water. After heating, without boiling, the alginate solution was filtered with a 0.22 µm Millex^®^-GP syringe filter (Merck KGaA, Darmstadt, Germany) to remove undissolved substances and impurities. Medium molecular weight chitosan from crab shells, 85% deacylated, (Sigma, St. Louis, MO, USA) was dissolved in deionized water with 2% glacial acetic acid, and twice-filtered to remove undissolved substances and impurities. This yielded a 2% (*w*/*v*), pH 5 solution of chitosan. A 1% (*w*/*v*) calcium chloride solution was prepared by dissolving calcium chloride dihydrate, CaCl_2_, (JT Baker, Phillipsburg, NJ, USA) in distilled water, and filtering. ATP, 100 mM, was obtained (Thermo Scientific Inc., Rockford, IL, USA) and diluted to 10 mM with autoclaved MilliQ water (Millipore, Darmstadt, Germany).

### 4.8. Calculating Bound SsoPox

Binding of enzymes to chitosan films and capsules was verified by labeling *Sso*Pox with DyLightTM sulfhydryl-reactive dye. The amount of chitosan in the bottom of the 96-well plate and surface area of the well was used to calculate amount of *Sso*Pox per surface area of chitosan. The outer diameter (1.375 mm) and shell thickness of capsules (0.2 mm) were measured immediately after rinsing using an MVX10 MacroView fluorescence stereomicroscope (Olympus, Center Valley, PA, USA). This information was used to calculate the average surface area of the capsule (23.76 mm^2^) and subsequent amount of bound *Sso*Pox based on the surface area of a well in a 96-well plate (0.32 cm^2^).

The chitosan film at the bottom of a 96-well plate was used initially to verify binding and estimate binding density. Labeled *Sso*Pox was incubated with the dried chitosan; a plate reader was used to take fluorescence readings during incubation and after rinsing. Using these post-rinse fluorescence readings, the amount bound was calculated.

In addition, the maximum amount of enzyme per surface area was calculated for each well using the molecular weight of *Sso*Pox (38.7, 39.6 kDa). For this work, a square packing hexagon model shown as 7 circles within a circle (see Figure S5), was used to estimate the quantity of protein that fills the projected surface area. Approximately 7/9 or 78% of the surface area is covered by a spherical protein. This corresponds to 1.9 pmol of *Sso*Pox that covers an area of 0.25 cm^2^ (7.6 pmol/cm^2^). The thickness of single layer of enzyme was estimated to ~ 4.5 × 10^−7^ cm.

## Supplementary Materials

The following are available online. Figure S1: Linear relationship between AI-1 concentration and luminescence, Figure S2: Linear ranges of two fluorescent reporters, Figure S3: Construction primers & Gene Block, Figure S4: AHL Reporter Plasmid Maps, Figure S5: Representative Spacing of Enzymes on a Surface, Figure S6: Bound, Labeled *Sso*Pox-Gln Comparison.
