# Supplementary figures and images for: Modification and Assembly of a Versatile Lactonase for Bacterial Quorum Quenching
Source: Molecules. 2018 Feb 6;23(2):341. doi: 10.3390/molecules23020341 (PMC6016966; doi:10.3390/molecules23020341)

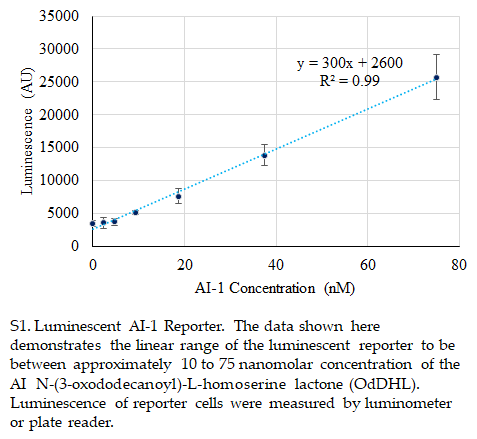

Supplement: Supplementary file 1 [file molecules-23-00341-s001.zip › Figure S1.tif]

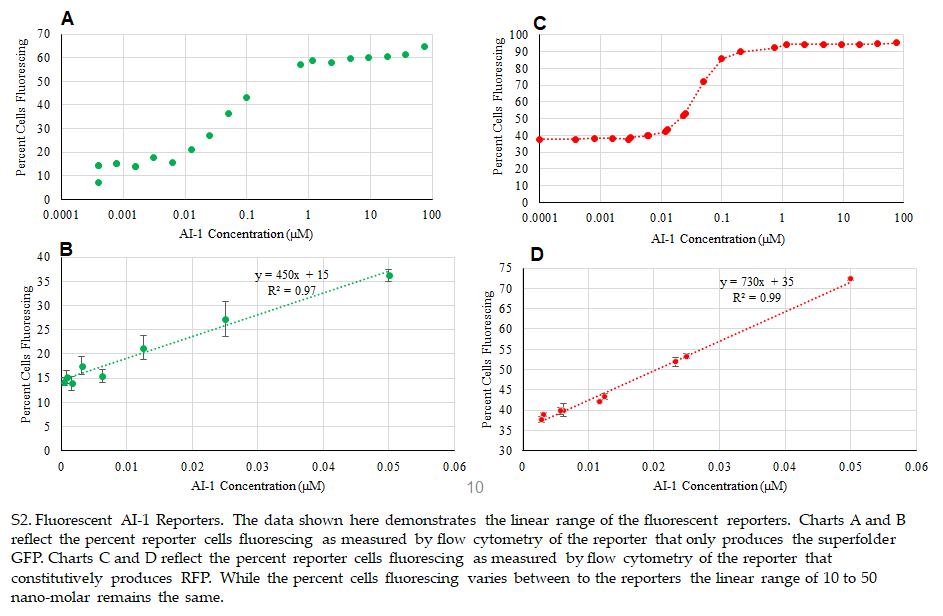

Supplement: Supplementary file 1 [file molecules-23-00341-s001.zip › Figure S2.tif]

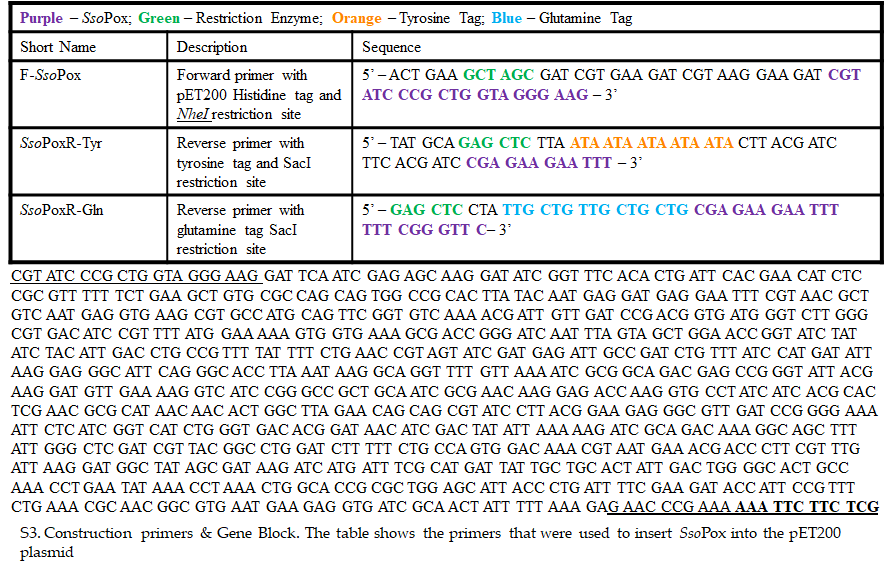

Supplement: Supplementary file 1 [file molecules-23-00341-s001.zip › Figure S3.tif]

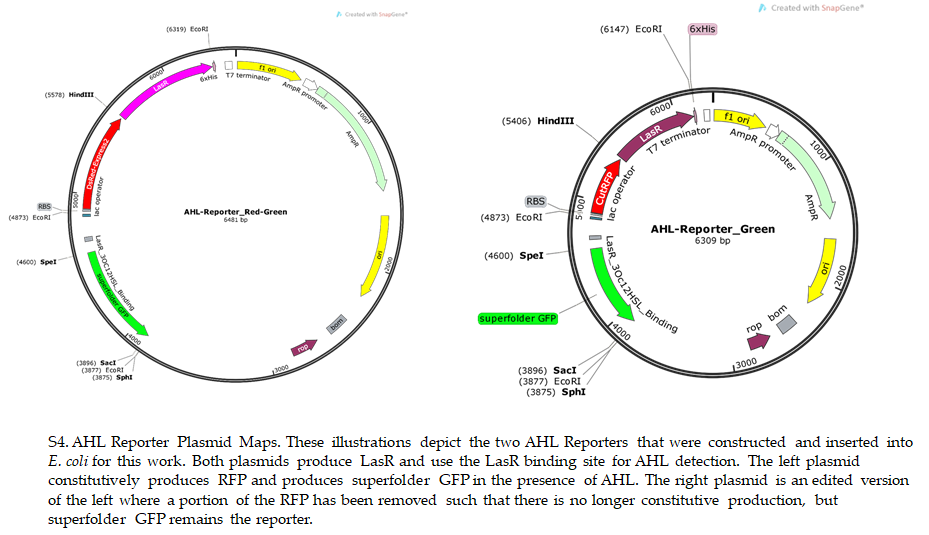

Supplement: Supplementary file 1 [file molecules-23-00341-s001.zip › Figure S4.tif]

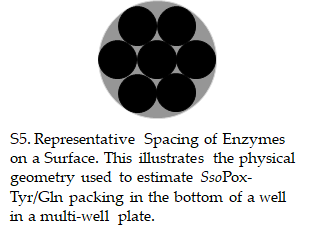

Supplement: Supplementary file 1 [file molecules-23-00341-s001.zip › Figure S5.tif]

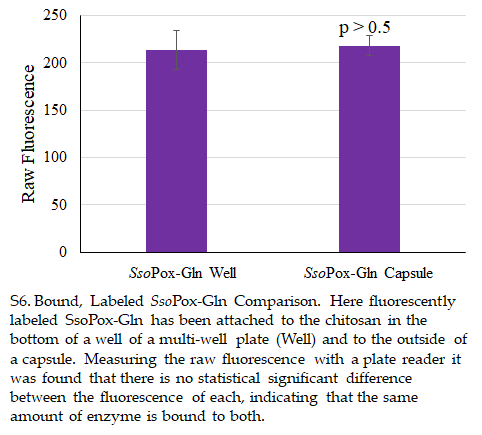

Supplement: Supplementary file 1 [file molecules-23-00341-s001.zip › Figure S6.tif]
